# Bioassay-Guided Procedure Coupled with HR-ESIMS Dereplication for Isolation of Antiproliferative Bromo-Tyramine Derivative from *Aplysina cauliformis*

**DOI:** 10.3390/md23050187

**Published:** 2025-04-27

**Authors:** Germana Esposito, Maria Ponticelli, Luigi Milella, Ludovica Lela, Roberta Teta, Joseph R. Pawlik, Daniela Russo, Valeria Costantino

**Affiliations:** 1 The Blue Chemistry Lab, Department of Pharmacy, University of Naples Federico II, Via Domenico Montesano 49, 80131 Napoli, Italy; roberta.teta@unina.it (R.T.); valeria.costantino@unina.it (V.C.); 2 Department of Biochemical Pharmacology & Drug Design, Institute of Molecular Biology “Roumen Tsanev”, Bulgarian Academy of Sciences (BAS), Acad. G. Bonchev Str., bl. 21, 1113 Sofia, Bulgaria; 3 Department of Health Science, University of Basilicata, Via dell’Ateneo Lucano 10, 85100 Potenza, Italy; luigi.milella@unibas.it (L.M.); ludovica.lela@unibas.it (L.L.); daniela.russo@unibas.it (D.R.); 4 Department of Biology and Marine Biology, Center for Marine Science, University of North Carolina Wilmington, 5600 Marvin K Moss Lane, Wilmington, NC 28409, USA; pawlikj@uncw.edu; 5 Spinoff BioActiPlant s.r.l., Via dell’Ateneo Lucano 10, 85100 Potenza, Italy

**Keywords:** *Aplysina cauliformis*, apoptosis, antiproliferative activity, *N*,*N*,*N*-trimethyl-3,5-dibromotyramine, marine bromo-tyramine analog, blue growth, blue bioeconomy

## Abstract

The marine environment is vital for sustaining life on Earth and offers a significant, untapped source of bioresources that could enhance the blue economy. The present investigation used our protocol to quickly identify bioactive molecules in *Aplysina cauliformis* organic extracts. This procedure combines a bioassay-guided approach with the dereplication of mass data through bioinformatic analysis. This approach identified the compound *N*,*N*,*N*-trimethyl-3,5-dibromotyramine, a bromo-tyramine analog that showed promising antiproliferative activity on HepG2 cell lines, with an IC_50_ value of 37.49 ± 1.94 μg/mL after 24 h. Furthermore, the evaluation of related gene expression confirmed the mechanism of cell death to be apoptosis. *N*,*N*,*N*-trimethyl-3,5-dibromotyramine increased the expression of pro-apoptotic *β*-cell lymphoma 2-associated X protein (BAX) and Poly (ADP-ribose) polymerase (PARP-1) cleavage (c-PARP-1) and downregulated the anti-apoptotic β-cell lymphoma 2 (BCL-2) and phospho-Akt (p-AKT). This report presents *N*,*N*,*N*-trimethyl-3,5-dibromotyramine from *Aplysina cauliformis* and its antiproliferative activity against the HepG2 cell line.

## 1. Introduction

Caribbean coral reefs are home to various sponges that dominate the benthic community, contributing significantly to its biomass and biodiversity. Our research group has been conducting in-depth studies on the chemistry of Caribbean marine sponges since 1992 as part of an international cooperation program focused on the chemistry and ecology of marine porifera (NSF Biological Oceanography 9806930, “Assessing the chemical defenses of Caribbean Invertebrates”).

This pioneering program has introduced the blue economy era, one of the pillars of the European Union’s long-term strategy to support sustainable growth in the marine and maritime field. It is goal number 14 among the 17 sustainable goals (SDGs) of the Agenda 2030 (https://www.un.org/sustainabledevelopment/development-agenda/, accessed on 18 December 2024).

Sponges have been primarily studied in the last thirty years. They are considered one of the richest sources of novel secondary metabolites, often possessing complex structures that enrich the chemodiversity of natural products [1,2,3]. Most show potent pharmacological properties, ranging from antimicrobial to anticancer [4,5,6]. The *Aplysina* genus (Order Verongida) includes large sponges that are different in form and color, and they are among the most common benthic animals that inhabit the Caribbean, showing vast biodiversity [7]. Caribbean sponges of the genus *Aplysina* are a rich source of brominated secondary metabolites, a series of tyrosine-derived brominated alkaloids present in all species in different amounts, supporting a robust taxonomic marker’s theory. This compound class protects the marine organism against predators and microorganisms [7,8]. In addition, several brominated analogs of tyramine have been detected. These compounds exhibit potent antiproliferative activities, with the bromine atoms enhancing lipophilicity and interaction with cell membranes, leading to effects like apoptosis induction and disruption of critical cellular processes.

Several brominated tyramines, such as 3,5-dibromotyramine, have exhibited promising cytotoxic activity against various cancer cell lines, including breast, colon, and ovarian cancers [9]. The bromination pattern, which includes substitutions at crucial positions on the aromatic ring, is believed to play a significant role in their bioactivity, particularly by improving interactions with membrane-bound proteins and intracellular targets [10].

Our ongoing drug discovery program utilizes a protocol to swiftly identify bioactive molecules through a bioassay-guided procedure, combined with dereplication of extracts based on bioinformatic analyses. This protocol has been validated by examining the organic extract from the marine sponge *Aplysina cauliformis*.

The primary objective of this study is to uncover novel bioactive secondary metabolites from marine sources, specifically focusing on Caribbean coral reefs and their resident sponges. By exploring the chemical diversity of marine organisms, we aim to identify compounds with promising pharmacological activities that can be developed for therapeutic purposes. The discovery of bioactive molecules from natural sources, such as the brominated alkaloids found in Caribbean sponges, holds immense potential for the development of new drugs, particularly in the fields of antimicrobial and anticancer therapies.

The use of molecular networking as a powerful dereplication tool was discovered in the bioactive extract of *A. cauliformis*, a cluster belonging to known brominated molecules probably responsible for the activity detected. This study, coupled with a bioassay-guided isolation procedure, led to the isolation of *N*,*N*,*N*-trimethyl-3,5-dibromotyramine **1**, a bromo-tyramine derivative, showing antiproliferative activity with an IC_50_ value of 37.49 ± 1.94 μg/mL on the HepG2 cell line. While related brominated tyramines and their analogs have demonstrated significant bioactivity, no specific studies on the cytotoxic potential of *N*,*N*,*N*-trimethyl-3,5-dibromotyramine have been reported [11,12].

This paper introduces a groundbreaking protocol for swiftly identifying bioactive molecules present in any natural organic extract.

## 2. Results

### 2.1. Bioassay-Guided Procedure Coupled with HR-ESIMS Dereplication for the Isolation of a Bioactive Compound from Aplysina cauliformis

A bioassay-oriented approach was used to assess the bioactivity of *A. cauliformis* extract using an MTT assay. The protocol used in our lab to evaluate its cytotoxic potential on hepatic cancer cells (HepG2 cells), compared with normal cells (IHH cells), demonstrated that the organic fraction has a moderate cytotoxic effect on HepG2 with an IC_50_ value of 214.29 ± 1.19 µg/mL. The organic fraction was fractionated using RP-C18 silica gel chromatographic techniques, leading to seven subfractions that were analyzed by LC-MS/MS and screened for their antiproliferative activity (see Table 1). Data obtained illustrated that the fraction named A4 showed the highest cytotoxicity (IC_50_ 134.28 ± 1.05 µg/mL) on HepG2 cells. The LC-MS/MS analysis of A4, B, and C fractions from RP-C18 was performed with an LTQ ORBITRAP XL mass spectrometer with an ESI ionization source (electrospray ionization). Data-dependent acquisition was employed to initiate MS2 scans for the five most intense ions detected in the full MS scan. This approach yielded high-resolution raw data, which underwent pre-processing steps using the MZmine software version 2.53 [13]. The goal was to filter LC-MS/MS data, identify isotopic peaks, and distinguish isomers based on varying retention times. Ultimately, this process aimed to achieve a molecular network with a unique node representing each compound, focusing specifically on selecting chlorinated or brominated metabolites. These metabolites are often the primary contributors to the extract’s activity. The processed files were then uploaded to the GNPS2 site [14] to generate the final network version (GNPS2—Analysis Hub), which was visualized using the Cytoscape program version 3.10.1 [15].

**Figure 1:**
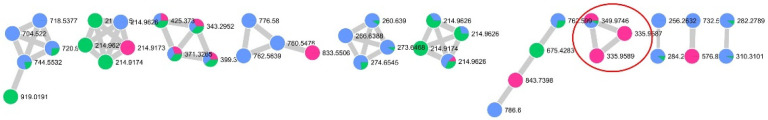
A portion of the molecular network of A4, B, and C fractions of RP-C18 chromatography. In the red circle is a cluster belonging to brominated compounds. Nodes are represented as a pie chart showing the source extract of the compound (fr. A4 (pink), fr. B (light blue), fr. C (green)).

A comparative network of A4, B, and C fractions from RP-C18 chromatography comprising 506 features grouped into 16 clusters was created. In the network, each node is depicted as a pie chart illustrating the compound’s abundance in A4 (pink), B (light blue), and C (green) fractions (Figure 1). Our analyses found that the only cluster containing all compounds from the bioactive A4 fraction had brominated compounds that could be responsible for the detected activity.

Specifically, we identified the node at *m*/*z* 335.9589 ([M]^+^, C_11_H_16_ONBr_2_^+^) as the *N*,*N*,*N*-trimethyl-3,5-dibromotiramine (**1**). The HR-MS/MS spectrum of this compound exhibited a characteristic fragment ion at *m*/*z* 276.8854 ([M+H]^+^ C_8_H_7_OBr_2_^+^), arising from the loss of the trimethylamine group. This known brominated alkaloid has never been studied for its cytotoxic properties. This sparked our interest in isolating it, testing it for its antiproliferative activity, and showing that it was responsible for the bioactivity of the extract.

The node at *m*/*z* 349.9745 ([M]^+^, C_12_H_18_ONBr_2_^+^) was instead identified as the homologous superior of the compound **1** with a characteristic fragment ion at *m*/*z* 290.9010 ([M+H]^+^ C_9_H_9_OBr_2_^+^) in the HR-MS/MS spectrum.

To identify the compound responsible for the activity, fraction A4 was further purified (see experimental). This resulted in 34 HPLC fractions being screened for cytotoxicity on HepG2 cells. The most cytotoxic HPLC fractions were fr. A4_HPLC 3 and fr. A4_HPLC 13, which underwent further chromatographic analysis.

**Figure 2:**
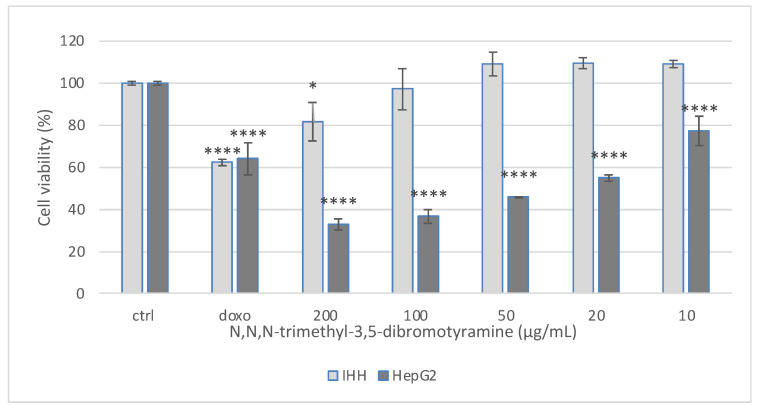
Cytotoxic effect of *N*,*N*,*N*-trimethyl-3,5-dibromotyramine on hepatic tumoral (HepG2) and Immortalized Human Hepatocyte (IHH) cell lines after 24 h of treatment. Doxorubicin (doxo 1 μM) was used as positive control. Data are expressed as the mean ± standard deviation of three independent experiments (n = 3) and were analyzed by one-way ANOVA followed by Tukey’s post hoc test. **** *p* ≤ 0.0001; * *p* ≤ 0.05 vs. untreated cells (CTRL).

Analysis of fr. A4_HPLC 3 confirmed the presence of compound (**1**) as a pure compound. In contrast, the analysis of fr. A4_HPLC 13 allowed for obtaining three subfractions, but only one of them reported significant cytotoxic effects on HepG2 cells (25.89 ± 1.94 µg/mL). The reduction in IC_50_ of some fractions compared to the pure compound may be due to synergistic effects between bioactive compounds of the fractions, as is well documented in natural product research [16,17]. Data of the cell viability on normal cell IHH allowed for continuing with the investigation of compound **1** (IC_50_ value higher than 200 µg/mL, 81.61% viability at 200 µg/mL, Figure 2), whereas all other fractions, despite their cytotoxicity on HepG2, were excluded from the study for the reduced viability of normal cells after treatment (IC_50_ < 20 µg/mL for IHH cells). Treatment with doxorubicin, an agent known to induce cell death, reduced viability by approximately 40%.

The compound has previously been isolated from *Aplysina fistularis*, *Verongula gigantea*, *Verongula rigid*, and *Aplysinella* sp. [18,19,20], and recently, it was found for the first time [21] in *Aplysina aerophobia*.

^1^H NMR and HR-ESIMS assessed the chemical structure of the active molecule **1**. In particular, the positive ion mode HR-ESIMS of **1** displayed a molecular ion peak [M]^+^ at *m*/*z* 335.9591 with an isotopic pattern in the ratio of 1:2:1, respectively, accounting for the presence of two bromine atoms and the molecular formula C_11_H_16_Br_2_NO^+^.

The ^1^H NMR proton spectrum showed a singlet counting for two aromatic protons at δ 7.50 and two multiplets at δ3.03 and δ3.52 corresponding to two mutually coupled methylenes in which each proton of one CH_2_ has different coupling constants with those of the other CH_2_, probably as a result of the presence of bulky groups such as the benzenic ring that cause the anti-conformation to be favored over the gauche, and a singlet counting for nine protons corresponding to the trimethyl group at δ3.19.

**Figure 3:**
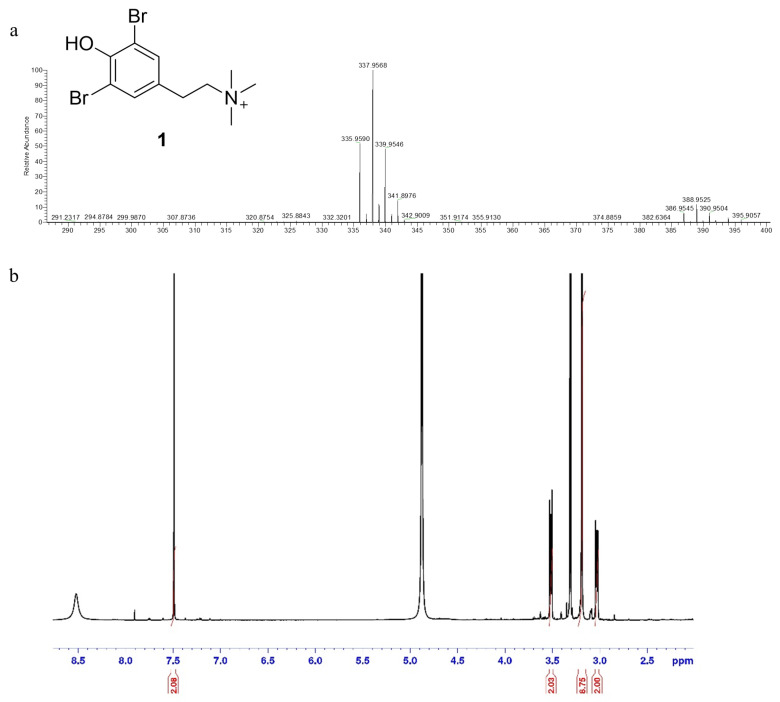
Compound **1**, *N*,*N*,*N*-trimethyl-3,5-dibromotyramine. (**a**) HR-ESIMS spectrum of compound **1**. (**b**) ^1^H spectrum of compound **1**.

Taken together, HR-ESIMS data and the comparison of the ^1^H-NMR data (Figure 3) with data reported in the literature allowed the assignment of the structure of compound **1** as *N*,*N*,*N*-trimethyl-3,5-dibromotyramine.

### 2.2. Effect of N,N,N-Trimethyl-3,5-Dibromotyramine on Apoptosis and Apoptotic Markers in HepG2 Cells

**Figure 4:**
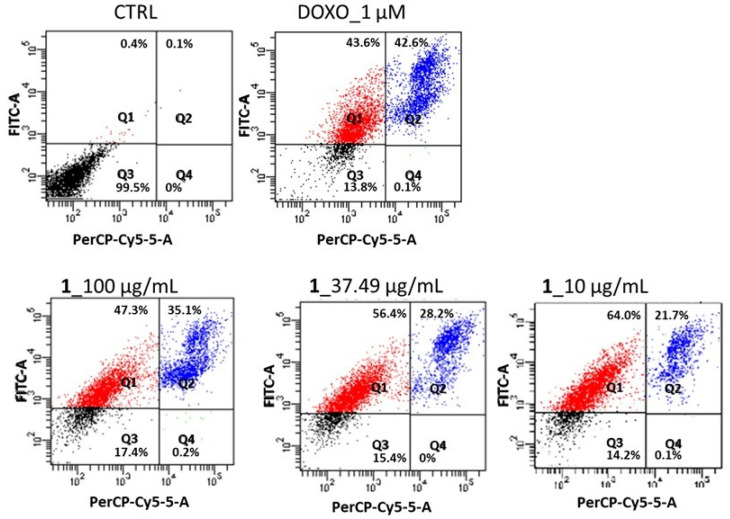
V-FITC/PI staining assay for apoptosis evaluation by flow cytometry. The HepG2 cells were treated with *N*,*N*,*N*-trimethyl-3,5-dibromotyramine (100, 37.49 and 10 µg/mL) after 24 h treatment. The data represent one of three independent experiments. Q1 quadrant shows the early apoptosis cells, Q2 quadrant shows the late apoptosis cells, Q3 quadrant shows the viable cells, and Q4 quadrant shows necrotic cells. CTRL, Control; DOXO, doxorubicin; **1**, *N*,*N*,*N*-trimethyl-3,5-dibromotyramine compound.

To investigate the reduced HepG2 cell growth, the effect of the *N*,*N*,*N*-trimethyl-3,5-dibromotyramine as an apoptosis-inducer was detected using flow cytometry and Annexin V-FITC/PI staining assay. The double staining allowed for discriminating between early and late apoptosis stages and necrosis. The significant feature of the early apoptosis stage is the translocation of phosphatidylserine to the external surfaces of the membrane, detected by annexin V-FITC binding. The binding with PI occurs when cells die, whereas the cell staining with annexin V and PI means that the cellular membrane has been damaged and they are in the late apoptosis stage. After 24 h of treatment, the quali–quantitative assessment of apoptosis for compound **1** at the IC_50_ value (37.49 ± 1.94 µg/mL), 10 µg/mL, and 100 µg/mL was carried out, and the cell distribution into four quadrants is reported in Figure 4.

As expected, the untreated cells reported the highest number of viable cells, 99.5 ± 0.13%, showing that the cells were still healthy. In the early apoptosis stage, the control contained 0.4 ± 0.02% of cells, whereas the treatment with the *N*,*N*,*N*-trimethyl-3,5-dibromotyramine increased the percentage of cells in this stage to 47.3%, 56.4%, and 64.0% at 100, 37.49, and 10 µg/mL, respectively. In the late apoptosis stage, the fraction of apoptotic cells increased from 21.7% (10 µg/mL) to 35.1% (100 µg/mL). Figure 3 shows the percentage of cells in the apoptotic stage of *N*,*N*,*N*-trimethyl-3,5-dibromotyramine. The doxorubicin was used as a positive control.

**Figure 5:**
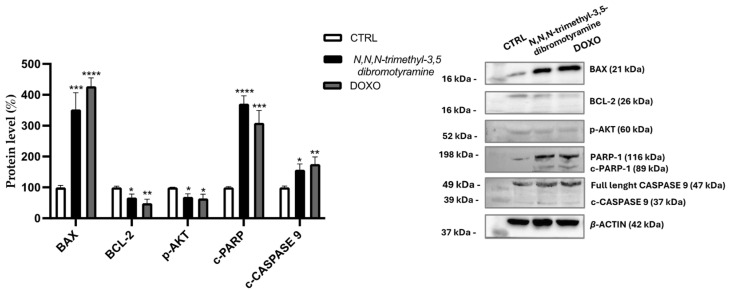
Effect of *N*,*N*,*N*-trimethyl-3,5-dibromotyramine on apoptotic marker expression in HepG2 cells after 24 h. Immunoreactive bands are from a single experiment representative of multiple experiments; the protein levels were normalized with *β*-actin content, and data were normalized to control cells set to 100%. One-way ANOVA followed by Tukey’s post hoc test was used for the analyses. **** *p* < 0.0001, *** *p* < 0.001, ** *p* < 0.01, * *p* < 0.05 vs. control cells (CTRL). DOXO: doxorubicin.

The Western blot was performed to investigate the molecular pathway involved in the induction of HepG2 cell apoptosis by *N*,*N*,*N*-trimethyl-3,5-dibromotyramine. The treatment with IC_50_ value (37.49 µg/mL) of pure compound and doxorubicin, used as positive control, increased the expression of cleaved CASPASE-9 and of the pro-apoptotic B-cell lymphoma 2-associated X protein (BAX) while it reduced the anti-apoptotic B-cell lymphoma 2 (BCL-2) after 24 h. Furthermore, the pure compound downregulated phospho-Akt (p-AKT) protein expression while inducing Poly (ADP-ribose) polymerase (PARP-1) cleavage (c-PARP-1), a biochemical hallmark of apoptosis (Figure 5).

## 3. Discussion

Numerous compounds with significant biological properties have recently been identified in marine sponges, but this field still holds promise in terms of chemical diversity and bioactivity.

The marine sponge *Aplysina cauliformis* (brown erected morphotype) collected along the coast of Great Inagua (Bahamas Islands) was extracted using MeOH/CHCl_3_ mixtures. Following our experimental protocol [22,23] for fast identification of bioactive molecules, the bioassay-guided procedure coupled with HR-ESIMS dereplication of extracts based on bioinformatic analyses was applied. This approach allows for the identification of compound 1 as *N*,*N*,*N*-trimethyl-3,5-dibromotyramine. This is the first evidence of *N*,*N*,*N*-trimethyl-3,5-dibromotyramine in *A. cauliformis*, which was previously found in *Aplysina fistularis* Pallas, *Verongula gigantea* Hyatt, *Verongula rigida* Esper, *Aplysinella* sp. [18,19,20], and recently from *Aplysina aerophoba* Nardo [21].

Bromotyramine derivatives, typical of *Aplysina* sponge species, have been found to possess a large chemical diversity and exhibit diverse activities, including antimicrobial, antifungal, antiviral, and anticancer [20]. Aeroplysinin-1 (1–5 µM) from *Aplysina aerophoba*, significantly reduced the viability of the neuroblastoma SH-SY5Y cell line [24], and aplysinin A from *Aplysina lacunosa* Lamarck showed a cytotoxic effect against the breast cancer cell line MCF-7 (IC_50_ 78 µM) [25]. A previous study investigated the effect of *N*,*N*,*N*-trimethyl-3,5-dibromotyramine from *A. aerophoba* on human stomach carcinoma (AGS) and human urinary bladder carcinoma (T24) cell lines, showing no toxicity at the tested concentration (100 µM) [21]. In this study, *N*,*N*,*N*-trimethyl-3,5-dibromotyramine showed marked toxicity (IC_50_ value of 37.49 µg/mL) after 24 h treatment; therefore, this molecule was selected for further investigation. Interestingly, no toxic effect was observed in the human hepatocyte cell line (IHH) at tested concentrations. Flow cytometric analysis by annexin V and PI double staining showed a significant increase in cell percentage in the apoptotic subpopulation from 0.4–0.1% (untreated cells) to 56.4–28.2% (IC_50_ treatment cells) in early–late apoptosis, respectively.

We found that cell death is linked to apoptotic mechanisms. Apoptosis, often called programmed cell death, is a highly regulated and controlled process through which cells undergo self-destruction in response to specific signals. This mechanism plays an essential role in eliminating damaged, unwanted, or potentially harmful cells. However, dysregulation affects numerous diseases, such as immunological disorders, neurodegenerative diseases, and cancer. Apoptosis is characterized by specific biochemical processes that involve different markers [26]. Key players in the regulation of apoptosis include proteins from the BCL-2 family, particularly BAX and BCL-2, as well as cleaved PARP (Poly ADP-ribose polymerase). There are two main apoptotic pathways, the extrinsic and intrinsic or mitochondrial, that are closely related to each other [27]. Mitochondria, the primary center for energy production and metabolism in cells, are crucial for regulating apoptosis signaling pathways. The regulation of mitochondrial events that characterize apoptosis occurs thanks to the BCL-2 family of proteins. In the present study, the pure compound *N*,*N*,*N*-trimethyl-3,5-dibromotyramine downregulated the expression of the anti-apoptotic BCL-2 localized in the outer mitochondrial membrane and, conversely, upregulated the pro-apoptotic BAX which is normally present in the cytosol but during apoptosis process is translocated on the mitochondrial membrane [28,29]. The balance between pro- and anti-apoptotic Bcl-2 family proteins plays a key role in maintaining mitochondrial membrane potential and determining whether a cell undergoes apoptosis [30]. An increase in the BAX/BCL-2 ratio improves membrane permeability due to a reduction in mitochondrial membrane potential, which causes cytochrome C release in the cytoplasm from the mitochondria. This is a key event in apoptosis. Cytochrome C enters the cytoplasm, where it binds to apoptosis protease activating factor 1 (APAF-1), initiating the formation of a complex, the apoptosome. This complex facilitates the binding of caspase-9 to APAF-1, leading to the formation of apoptotic bodies [31]. This induces apoptosis by activating some caspases, such as caspase-3 or 9, and promoting the degradation of PARP. Indeed, in our study, the expression of cleaved caspase 9 and cleaved PARP-1 after treatment was increased compared with untreated cells. As shown in the apoptosis dot plots, the presence of viable cells in both early and late stages of apoptosis indicates a transitional phase in the population. During this phase, an increase in the expression of full-length PARP in response to DNA damage could be observed as well as an increase in cleaved PARP via activated caspases, leading to the accumulation of cleaved PARP fragments.

Activation of AKT signaling counteracts both intrinsic and extrinsic pathways of apoptosis. Downregulation of the expression of the phosphorylated form at Ser473 within 24 h of treatment with the pure compound reduced its cell protective effect against apoptosis [32].

Many drugs used in cancer treatment do not show high specificity and often show toxic effects, so new alternatives are necessary [33]. Our findings showed that *Aplysina cauliformis* is a source of compounds with potential health benefits, and in particular, the presence of *N*,*N*,*N*-trimethyl-3,5-dibromotyramine represents a potential anticancer agent.

## 4. Materials and Methods

### 4.1. General Experimental Procedures

HR-ESIMS experiments were performed on a Thermo LTQ Orbitrap XL mass spectrometer (Thermo Fisher Scientific Spa, Rodano, Italy) coupled to an Agilent model 1100 LC system (Agilent Technology, Cernusco sul Naviglio, Italy). The spectra were recorded by infusion into the ESI source using MeOH as the solvent. NMR spectra were determined at 700 MHz on a Bruker Avance Neo spectrometer (Bruker BioSpin Corporation, Billerica, MA, USA); chemical shifts were referenced to the residual solvent signal (CD_3_OD: δH 3.31, δC 49.00). High-performance liquid chromatography (HPLC) separations were achieved on an Agilent 1260 Infinity Quaternary LC apparatus (Agilent Technology, Cernusco sul Naviglio, Italy) equipped with a diode-array detector (DAD).

### 4.2. Marine Sponge Material

*Aplysina cauliformis* Carter, a marine sponge weighing 277 g, was collected from the Great Inagua coast of the Bahamas (21°03024.5500 N, 73°25027.7600 W) at a depth of 15 m on 7 July 2013. The collection was conducted via scuba diving, aiming to gather small amounts of the sample while minimizing impact on the organism. Following collection, the sponge was promptly identified onboard using information from The Sponge Guide (www.spongeguide.org). It was then frozen immediately and stored at −20 °C until extraction. A voucher specimen is preserved at the “Blue Chemistry Lab” in the Dipartimento di Farmacia at the Università degli Studi di Napoli “Federico II”, with the reference number 07/07/13.

### 4.3. Extraction and Isolation

The marine sponge *Aplysina cauliformis* (277 g), after reaching room temperature, was cut into small pieces, homogenized, and extracted by MeOH (3 × 400 mL), MeOH/CHCl_3_ 2:1 (3 × 400 mL), MeOH/CHCl_3_ 1:2 (3 × 400 mL), and CHCl_3_ (3 × 400 mL) giving 8.5 g, 0.659 g, 0.108 g, and 0.082 g of extract, respectively. An aliquot of MeOH extract (4 g) was solubilized in water and partitioned using n-BuOH (1.70 g). The organic fraction was mixed with CHCl_3_ extracts, defined here as all fractions obtained using CHCl_3_-containing solvents, including CHCl_3_ alone as well as MeOH/CHCl_3_ mixtures in different proportions, to yield the BuOH-CHCl_3_ fraction (2.01 g), which was then subjected to column chromatography on RP-C18 Silica gel. The fraction was eluted with MeOH/H_2_O 1:9 (A1, 79.66 mg), 3:7 (A2, 328.87 mg), 6:4 (A3, 801.08 mg), 8:2 (A4, 173.07 mg), 9:1 (A5, 18.34 mg), 9:1 (A5*, 11.6 mg), MeOH/ CHCl_3_ 9:1 (B, 328.92), and finally 100% CHCl_3_ (C, 28.94 mg). An aliquot of each fraction was used to evaluate bioactivity; the other part was preserved for purification.

The aliquot of fraction eluted with MeOH/H_2_O (8:2, A4) (95.7 mg) was subjected to reversed-phase HPLC separation (column 250 × 10 mm, 10 μm, Luna (Phenomenex) C18; Eluent A: H_2_O + 0.1%HCOOH; Eluent B: MeOH; gradient: 10%→100% B over 20 min, flow rate 5 mL/min), thus affording a fraction (*t*R = 8.2 min) containing pure compound **1**.

Compound **1** HR-ESIMS [M]+ *m*/*z* 335.9590, 337.9568 and 339.9547 for C_11_H_16_Br_2_NO^+^ calcd 335.9599, 337.9578 and 339.9558; ^1^HNMR (700 MHz, CD_3_OD): δ 7.50 (2H, s), δ 3.51 (2H, m), δ 3.03(2H, m), δ 3.19 (9H, s, N-(CH_3_)_3_).

### 4.4. LC-HR-ESIMS and LC-HR-MS/MS Analysis

LC-HR-ESIMS and LC-HR-MS/MS analyses were conducted on a Thermo LTQ Orbitrap XL high-resolution mass spectrometer (ESI source), connected to an Agilent 1100 LC system. The system included a solvent reservoir, an inline degasser, a binary pump, and a cooled autosampler. The separation was performed on a Kinetex C18 column (5 µm, 50 × 2.1 mm) at ambient temperature, with a 200 µL/min flow rate. The mobile phase consisted of H_2_O with 0.1% formic acid and MeOH, following a gradient of 10% MeOH for the first 3 min, increasing to 100% over 30 min, and maintained at 100% for an additional 7 min. The injection volume was 5 µL. Data acquisition used positive ion mode, with settings including a spray voltage of 4.8 kV, a capillary temperature of 285 °C, sheath gas at 32 units (approx. 150 mL/min N_2_), and auxiliary gas at 15 units (approx. 50 mL/min N_2_). The instrument operated in data-dependent acquisition (DDA) mode, targeting the five most intense ions per full-scan mass spectrum for HR-MS/MS analysis within an *m*/*z* range of 150–2000 amu. Fragmentation employed CID with an isolation width of 2.0, a collision energy of 35%, an activation Q of 0.250, and a duration of 30 ms. Thermo Xcalibur software version 2.2 was used for data analysis.

### 4.5. LC-HRMS/MS Data Processing and Molecular Networking

Raw files were imported and processed using MZmine 2.53 software [13]. The data were initially cropped to narrow the *m*/*z* range between 200 and 2000 amu. Mass detection was performed on the cropped data, applying a noise threshold of 10,000 for both centroided mass levels 1 and 2. Chromatograms were generated with the ADAP module, using a minimum peak height of 10,000 and an *m*/*z* tolerance of 0.001 (5 ppm). Peak alignment was conducted using the Join aligner algorithm with an *m*/*z* tolerance of 0.005 (5 ppm) and a retention time (RT) tolerance of 0.2 min. Specific adducts, including [M+Na–H], [M+K–H], [M+Mg−2H], [M+NH_3_], [M+1, ^13^C], [M-^35^Cl+^37^Cl], [M+^56^Fe-3H], and [M-^79^Br+^81^Br], were filtered by setting their maximum relative heights at 100%. Isotopic peaks for compounds containing two bromine atoms ([M-^79^Br+^81^Br]) were filtered with a 400% relative height threshold. Peaks lacking MS/MS spectra were excluded. Feature-based data were exported to an .mgf file for GNPS2 analysis, while chromatographic details (retention times, peak areas, and heights) were saved as a .csv file. A Feature-Based Molecular Network [34] was created on GNPS2 [14] using parameters like a 0.05 Da precursor ion tolerance, 0.05 Da MS/MS fragment ion tolerance, a cosine score of ≥0.7, and at least 6 matching peaks. Only nodes within the top 10 most similar spectra were included. The same cosine score and peak match criteria were applied for library searches, and the network visualization was performed using Cytoscape software version 3.10.1 [15].

### 4.6. Cell Culture and Cytotoxicity Assay

Human hepatocellular carcinoma cell line (HepG2) cells were provided by American Type Culture Collection (ATCC) and cultured in DMEM supplemented with 10% fetal bovine serum, 2 mM glutamine, 100 U/mL penicillin, and 100 µg/mL streptomycin, while the Immortalized Human Hepatocyte (IHH) cells were maintained in DMEM F-12 supplemented with 10% FBS, 1% of 100 IU/mL penicillin, 100 μg/mL streptomycin, 1 μM dexamethasone, and 10^−12^ M insulin. Both cell lines were maintained at 37 °C in a humidified atmosphere containing 5% CO_2_.

Cell viability was assessed through the MTT assay, a colorimetric method that relies on the ability of living cells’ succinate dehydrogenase enzyme to turn the yellow tetrazolium salt MTT into a purple, insoluble formazan product. HepG2 cells were plated onto a 96-well plate (1.5 × 10^4^ cells per well) and incubated overnight. Subsequently, they were exposed to different concentrations of the fractions (ranging from 10 to 200 µg/mL) for 24 and 48 h. Following the treatment, the medium was discarded, and the cells were rinsed with PBS, followed by a 4 h incubation with MTT solution (0.75 mg/mL in PBS). Afterward, the MTT solution was removed, and a lysis buffer (a 50:50 mixture of DMSO and isopropanol with 1% TritonX-100) was added to dissolve the cells and the formazan crystals. The dissolved formazan absorbance was measured at 560 nm using a UV–Vis spectrophotometer (SPECTROstarNano BMG Labtech, Ortenberg, Germany) [35].

### 4.7. Apoptosis Analysis by Flow Cytometer

Sponge fractions or pure compounds were used to assay apoptosis in cancer cells by using Annexin V-FITC Apoptosis Detection Kit (Sigma Aldrich, Saint Louis, MO, USA). The cells, seeded in 24-well plates, were treated without and with compound **1** (100, 37.49, 10 μg/mL), and after 24 h, they were collected and suspended in the buffer. The mixtures were analyzed using the flow cytometry instrument after 30 min, following the manufacturer’s instructions. Flow cytometric analysis was carried out using a FACS Canto II Becton–Dickinson (Franklin Lakes, NJ, USA) flow cytometer by analyzing at least 5000 cells per sample [36]. Unstained cells and single-stained controls were used to set up the quadrants in the experiment. As reported by kit instructions, unstained cells were classified as viable cells; cells stained for Annexin-V were early apoptotic; cells stained with both Annexin-V and PI were late apoptotic; cells stained with PI were indicated as necrotic cells. Results are presented as percentage (%) of the total number of cells. Doxorubicin (1 µM) was used as the positive control.

### 4.8. Western Blot Analysis

HepG2 were seeded in a 6-well plate (8 × 10^5^ cells/well) and treated with 37.49 µg/mL (IC_50_) of *N*,*N*,*N*-trimethyl-3,5-dibromotyramine or 1 µM doxorubicin (doxo, an agent known to induce apoptosis) for 24 h. Changes in intracellular protein levels were evaluated by Western blotting. Cell lysates were prepared using RIPA buffer (0.2% SDS, 50 mM Tris–HCl pH 8, 1% sodium deoxycholate, 150 mM NaCl, 1% Igepal) supplemented with a phosphatase and protease inhibitors cocktail. After centrifugation, cell supernatants were collected, and protein content was quantified by Bradford assay [36]. The supernatant was resuspended in Laemmli sample buffer (60 mM Tris–HCl pH 6.8, 10% glycerol, 2% SDS, 1% dithiothreitol, and 0.002% bromophenol blue), and equal amounts of proteins were subjected to 12% SDS-PAGE gels (*w/v*) and transferred to nitrocellulose membranes. The membranes were incubated overnight with primary antibody in 5% skim milk at 4 °C. Anti-BCL-2 (1:1000) and anti-BAX (1:1000) (BioLegend), anti-*β*-ACTIN (1:5000) (ThermoFisher Scientific, Milan, Italy), anti-PARP-1 (1:1000), and anti-CASPASE 9 (1:1000) and anti-p-AKT (SER473) (1:750) (Cell Signaling) were used as primary antibodies. The primary antibodies were captured with suitable peroxidase-conjugated secondary antibodies for 1 h at room temperature. The immunoreacting bands were visualized by ECL™ Western Blotting Detection Reagents (GE Healthcare, Chicago, IL, USA) or the SuperSignal™ West Femto Maximum Sensitivity Substrate (Thermo Scientific), using Chemidoc TM XRS detection system equipped with Image Lab Software version 5.2.1 for image acquisition (Bio-Rad, Hercules, CA, USA). Densitometric analysis was performed by using Image J software version 1.53k. The protein expression level in the control sample was taken as 100%. Results were expressed as percentage of the value in comparison to the control sample.

### 4.9. Statistical Analysis

Biological data were expressed as mean ± standard deviation (Mean ± SD). Statistical analysis was performed using GraphPad Prism 8 Software, Inc. (San Diego, CA, USA), and *p* values ≤ 0.05 were considered as statistically significant.

## 5. Conclusions

The marine environment plays a critical role in sustaining life on Earth and offers a vast, untapped source of bioresources that can lead to novel drug discoveries. Multiple developments are required to unlock this hidden potential and conduct sustainable biodiscovery from marine organisms. Sustainable use of marine resources needs microanalysis techniques that minimize the extensive collection and use of large amounts of solvents. In this direction, this study proposed a bioassay-guided approach to isolating the cytotoxic compound from *A. cauliformis* marine sponge coupled with an HR-ESIMS dereplication strategy, allowing fast identification of the bioactive dibromotyramine.

The crude methanolic extract was found to reduce the viability of HepG2 cancer cells, leading to further fractionation and isolation of its components. All fractions, subfractions, and the isolated compound were screened for their cytotoxic activity against both HepG2 cells and Immortalized Human Hepatocytes (IHHs). From the active fraction, compound 1, identified as *N*,*N*,*N*-trimethyl-3,5-dibromotyramine, was isolated for the first time from the marine sponge *A. cauliformis*. This compound showed significant anticancer effects by triggering apoptosis in liver cancer cells and modifying the expression of apoptotic markers. These data support the selection of *A. cauliformis* as a natural source for anticancer drug discovery. Future studies will focus on analyzing the metagenome to verify whether the compound is produced by symbiotic microorganisms and to identify the gene responsible for its production. This will pave the way for sustainable production of bioactive compounds and their eco-friendly exploitation in the market. Moreover, it will be interesting to fully understand the potential of *N*,*N*,*N*-trimethyl-3,5-dibromotyramine as an anticancer agent, by conducting structure–activity relationship (SAR) studies and evaluating its mechanism of action in more depth and using other study models. This study underscores the importance of preserving marine biodiversity, as it is a source of valuable compounds with the potential to address global health challenges. Additionally, through the use of advanced techniques such as bioassay-guided isolation and molecular networking, we strive to streamline the process of identifying and characterizing bioactive compounds, contributing to the broader field of drug discovery from natural products.

## Figures and Tables

**Table 1:** Cytotoxic effect (IC_50_, μg/mL) of extract, fractions, and pure compound derived from *Aplysina cauliformis* marine sponge on HepG2 cells.

Extract	IC_50_ (µg/mL)
**MeOH**	237.99 ± 2.04 ^a^
**Partitioning fractions**	
***A. cauliformis* ** **ex MeOH R/H_2_O**	n.d.
*A. cauliformis* ex MeOH R/BuOH—CHCl_3_	214.29 ± 2.06 ^b^
**RP-C18 Silica gel CC fractions**	
fr. A1	n.d.
fr. A2	n.d.
fr. A3	292.42 ± 2.23 ^c^
fr. A4	134.28 ± 1.82 ^d^
fr. A5	234.42 ± 1.34 ^a^
fr. A5 *	204.17 ± 1.89 ^e^
fr. B	218.78 ± 2.12 ^b^
fr. C	202.77 ± 2.10 ^e^
**HPLC fractions from fr. A4**	
**fr. A4_HPLC 3 (pure compound)**	37.49 ± 1.94 ^f^
fr. A4_HPLC 4	559.24 ± 3.36 ^g^
fr. A4_HPLC 9	n.d.
fr. A4_HPLC 13	44.35 ± 2.06 ^h^
fr. A4_HPLC 19	236.66 ± 2.14 ^a^
fr. A4_HPLC 20	245.89 ± 2.09 ^i^
fr. A4_HPLC 22	n.d.
fr. A4_HPLC 28	277.24 ± 2.45 ^j^
fr. A4_HPLC 28 *	n.d.
fr. A4_HPLC 30	n.d.
**HPLC fractions from fr. A4_HPLC 13**	
fr. A4_HPLC 13/fr.9_HPLC 20/05/2021	n.d.
fr. A4_HPLC 13/fr.11_HPLC 20/05/2021	n.d.
fr. A4_HPLC 13/fr.13_HPLC 20/05/2021	25.89 ± 1.94 ^k^

Results were expressed as mean ± standard deviation (*n* = 3). Different letters indicate significant differences (*p* ≤ 0.05). n.d.= not detected. IC_50_ = Half maximal inhibitory concentration. * This is an additional little fraction near fr. 28

## Data Availability

The original data presented in the study are included in the article; further inquiries can be directed to the corresponding author.
